# Endocrine control of canine mammary neoplasms: serum reproductive hormone levels and tissue expression of steroid hormone, prolactin and growth hormone receptors

**DOI:** 10.1186/s12917-015-0546-y

**Published:** 2015-09-15

**Authors:** Michèle Spoerri, Franco Guscetti, Sonja Hartnack, Alois Boos, Christine Oei, Orsolya Balogh, Renata M Nowaczyk, Erika Michel, Iris M Reichler, Mariusz P Kowalewski

**Affiliations:** Clinic of Reproductive Medicine, Vetsuisse Faculty University of Zurich, Winterthurerstrasse 260, Zurich, 8057 Switzerland; Institute of Veterinary Anatomy, Vetsuisse Faculty University of Zurich, Winterthurerstrasse 260, Zurich, 8057 Switzerland; Institute of Veterinary Pathology, Vetsuisse Faculty University of Zurich, Winterthurerstrasse 268/272, Zurich, 8057 Switzerland; Section of Epidemiology, Vetsuisse Faculty, Winterthurerstrasse 270, Zurich, 8057 Switzerland; Department of Animal Health, Faculty of Veterinary Medicine, Utrecht University, P.O Box 80125, Utrecht, 3508 TC The Netherlands; Division of Animal Anatomy, Department of Animal Physiology and Biostructure, Faculty of Veterinary Medicine, Wroclaw University of Environmental and Life Sciences, Wroclaw, Poland

**Keywords:** Dog, Mammary neoplasm, Sex hormones, Hormone receptors

## Abstract

**Background:**

Neoplasms of the mammary gland are among the most common diseases in female domestic dogs (*Canis familiaris*). It is assumed that reproductive hormones influence tumorigenesis in this species, although the precise role of the endocrine milieu and reproductive state is subject to continuing discussion. In line with this, a recent systematic review of available data on the development of mammary neoplasms revealed weak evidence for risk reduction after neutering and an effect of age at neutering. Investigation of several hormone receptors has revealed decreased expression of estrogen receptor-alpha (ERα, ESR1), progesterone (P4) receptor (PGR), prolactin (PRL) receptor (PRLR) and growth hormone receptor (GHR) associated with neoplastic differentiation of mammary tissues. In other studies, increased levels of estrogens, progesterone and prolactin were found in serum and/or tissue homogenates of dogs with malignant neoplasms. However, the association between these entities within one animal population was never previously examined. Therefore, this study investigated the association between circulating serum concentrations of estradiol-17β, progesterone and prolactin, and gene expression of ERα (ESR1), ERβ (ESR2), PGR, PRLR, PRL and GHR, with respect to reproductive state (spayed vs. intact) and cycle stage (anestrus vs. diestrus). Additionally, the expression of E-cadherin (CDH-1) was evaluated as a possible indicator of metastatic potential.

**Results:**

For all receptors, the lowest gene expression was found in malignant tumors compared to normal tissues of affected dogs. Steroid levels were not influenced by their corresponding receptor expression in mammary neoplasms, but increased PRL levels were negatively associated with low PRLR gene expression in malignant tumors. The expression of CDH-1 was influenced by tumor malignancy and cycle stage, *i.e*., the highest gene expression was found in benign mammary tumors in diestrous dogs compared to normal and malignant mammary tissues of anestrous and spayed dogs.

**Conclusions:**

Herein, it has been confirmed that transformation towards malignant neoplasms is associated with significant reduction of gene expression of particular hormone receptors. Only PRLR in malignant tumors seems to be influenced by circulating PRL levels. In dogs, CDH-1 can be used as a prognostic factor; its expression, however, in benign tumors is influenced by cycle stage.

**Electronic supplementary material:**

The online version of this article (doi:10.1186/s12917-015-0546-y) contains supplementary material, which is available to authorized users.

## Background

Estrogens (estradiol 17β; E2), progesterone (P4), prolactin (PRL) and growth hormone (GH) are essential for physiological mammary growth and development, which not only take place during pregnancy but also occur with every reproductive cycle. While estrogens induce ductal growth, P4 is essential together with PRL for lengthening and tertiary branching of the ductal system and stimulation of lobulo-alveolar development [1, 2]. Furthermore, PRL promotes changes of the alveolar cells towards a secretory type and induces lactation [1–4]. The effects of these hormones are mediated through binding to their respective receptors within the mammary gland. Changes in the expression of E2, P4 and GH receptors (ER, PGR and GHR, respectively) but not of prolactin receptors (PRLR) have been described in normal canine mammary tissue during different phases of the estrous cycle [5–8]. In addition to physiological changes in the mammary gland during the estrous cycle, different pathological changes may occur. Tumors are considered to be the most common disorders of the mammary gland in dogs. Reproductive state and the endocrine milieu seem to play a pivotal role in the development of canine mammary tumors (CMT). The sparing effect of early spaying on CMT formation has been known since the work of Schneider and colleagues in 1969 [9]. However, a recent systematic review of peer-reviewed journal articles concluded that the evidence for a reduced CMT risk following ovariohysterectomy, as well as for an influence of age at neutering, is weak [10]. Studies investigating the impact of spaying, performed concurrently or shortly before the time of mammary tumor surgery, on disease-free interval or survival time yielded inconsistent results, showing either a beneficial effect of ovariohysterectomy or no effect [11–15]. Yet, a role for E2 in mammary tumorigenesis was suggested by its higher blood levels in dogs with malignant neoplasms compared to those without tumors [16–18]. On the other hand, bitches with inflammatory mammary carcinoma had lower estrogen levels compared to animals with other carcinoma subtypes [16]. An involvement of P4 in mammary tumorigenesis was also suggested, because treatment with progestins increased the risk of CMT [19–21], which is probably due to up-regulation of local GH production within the gland [22]. However, no significant differences in endogenously derived P4 serum concentrations were found between bitches with and without mammary neoplasms [17, 18]. A tumorigenic effect of PRL on the mammary tissue was hypothesized, because dogs with CMT had higher PRL levels than healthy animals [17, 23].

Despite the presumed influence of reproductive hormones, studies on endocrine therapy for CMT are rare. Gene or protein expression of ERα (ESR1) and/or ERβ (ESR2), PGR, PRLR and GHR were detected in CMTs, and decreased receptor expression was associated with malignancy and/or a worse prognosis [8, 16, 24–32]. Nevertheless, the application of a PGR blocker decreased PGR expression in canine mammary carcinoma cells and reduced cell proliferation *in vivo* and cell viability *in vitro* [33, 34]. The selective ER modulator tamoxifen, which is part of the standard therapy for women with ER-positive breast cancer [35–37], is not recommended in dogs due to its partial agonistic potential and the associated side effects [38, 39]. Lowering PRL serum levels by oral application of the dopamine receptor agonist cabergoline reduced the size of certain mammary lesions in 25 % of clinically pseudopregnant dogs presenting with mammary tumors [40].

Markers of tumor invasion are used in human medicine to estimate survival time and prognosis [41]. E-cadherin, the product of the CDH-1 gene, is expressed on the surface of most epithelial cells and regulates cell-cell adhesion. In women, loss or down-regulation of CDH-1 in breast carcinoma is associated with shorter survival time [42–44]. Similarly, in dogs, reduced immunostaining for CDH-1 is correlated with increased invasion potential, lymph node metastasis, histological grade and infiltration [45–48].

Mastectomy is regarded as the most effective treatment for CMT so far. However, further therapeutic options should be sought, which could be used in combination with surgery or in cases of non-operable lesions and inflammatory mammary carcinomas. Knowledge of the interplay between hormones and their receptors during the reproductive cycle, after spaying or during tumorigenesis, could provide a basis for new and advanced therapeutic approaches for dogs with CMT.

Up to now, there is no information on the effect of serum steroid hormone and PRL levels on their respective receptor gene expression in normal and neoplastic mammary tissues in dogs. Our goal was, therefore, to study the associations between circulating serum concentrations of E2, P4 and PRL, and the expression of genes encoding for their respective receptors, *i.e*., ERα (ESR1), ERβ (ESR2), PGR, PRLR, PRL, as well as GHR, in normal mammary tissues as well as in mammary neoplasms in female dogs, taking into account their reproductive state and cycle stage. Furthermore, gene expression of CDH-1 was analyzed as a marker for tumor invasiveness in mammary tissues showing different degrees of malignancy. In addition, the influence of reproductive state and cycle stages were assessed for all parameters.

## Methods

### Animals, tissues

A prospective study was carried out with 32 privately owned female dogs that were presented with mammary neoplasms to the Clinic of Reproductive Medicine, Vetsuisse Faculty in Zurich between 2009 and 2013. Altogether, 56 mammary neoplasms and 28 normal mammary tissue samples were collected from these patients. Eleven dogs had only one mammary neoplasm and 21 bitches had two or more lesions. Mastectomy was performed in 28 dogs to remove the masses. The following surgery techniques were used: unilateral mastectomy (n = 12), unilateral mastectomy combined with regional mastectomy (n = 3), regional mastectomy (n = 11), excisional biopsy (n = 2). Three of four dogs with inoperable cancer were euthanized at initial presentation because of their poor general condition. The fourth dog was euthanized two months later because of severe dyspnea due to pulmonary metastasis.

The phase of the estrous cycle at presentation was determined based on history, clinical examination and blood hormone assays (P4 and E2).

All owners consented to their animal becoming a research participant and animal experimentation was approved by the Cantonal Veterinary Authority in Zurich, Switzerland (permission No. 136/2009 and 165/2012).

### Blood sampling and hormone analysis

In order to account for the possible pulsatile release and circadian rhythm of PRL secretion [49, 50], two blood samples were collected 30 min apart and always in the morning from each dog prior to surgery or before euthanasia. Samples were centrifuged after clotting, and serum was stored at −20 °C until hormone analysis.

Serum PRL concentrations were measured with a previously validated heterologous radioimmunoassay (RIA) [51]. The intra-assay coefficient of variation was 3.5 %, and the lower limit of detection was 0.8 ng/ml. All serum samples were analyzed in the same run. The mean of the two PRL concentrations from each dog was used for statistical analysis.

E2 and P4 were determined from the first serum sample. E2 concentrations were determined by a solid-phase ^125^I-RIA (Count-A-Count TKE; Siemens Medical Solution Diagnostics, Los Angeles, CA, USA) according to the manufacturer’s instructions, with slight modifications as described previously and validated for the dog [52, 53]. The intra-assay coefficient of variation was 14 %, and the lower limit of detection was 1.9 pg/ml. Serum P4 concentrations were measured with a previously validated ^3^H-RIA [51, 54]. The intra- and inter-assay coefficients of variation were 11 and 9.7 %, respectively, and the lower limit of detection was 0.04 ng/ml.

### Tissue collection, histopathological classification and sample processing

Neoplastic and normal mammary tissue samples from each dog were collected by surgical mastectomy, biopsy or immediately post mortem. Tissue samples were fixed in 10 % buffered formalin for 24 h and embedded in paraffin. Sections from each block were stained with hematoxylin and eosin (HE) for histopathological diagnosis according to the criteria of Goldschmidt [55], and then grouped as normal mammary tissue, or benign or malignant mammary neoplasms.

The experimental procedure was based on our previously established protocol [25]. Thus, on the HE stained slides, representative parts of mammary neoplasms and normal mammary tissue from each dog were identified and marked. These parts were identified in the original paraffin blocks, cut out manually and re-embedded separately in paraffin. Consecutive sections from these new paraffin blocks were cut using a rotary microtome (RM 2165, Leica, Wetzlar, Germany). The first section (3 μm thick) was used for control HE staining, the following sections (10 μm thick) for RNA extraction (see below), and the last section (3 μm thick) for another control HE staining. The number of sections cut for RNA extraction varied from 15 to 30 depending on neoplasm size. The first and last control HE sections were compared to the selected representative part of the mammary tissue sample. If the control slides did not match the selected tissue, the original paraffin block was re-sampled and re-embedded as described above until both control slides matched the selected area.

### RNA extraction and semi-quantitative real-time (TaqMan) PCR

Excess paraffin was manually removed from each representative tissue section in a warm water bath (37 °C), and all sections were transferred into a 1.5 ml plastic Eppendorf tube (Eppendorf, Hamburg, Germany). Total RNA was extracted using the RNeasy FFPE Kit (Qiagen, Hombrechtikon, Switzerland) according to the manufacturer’s instructions. The RNA concentration was measured with a NanoDrop 2000 UV–Vis  Spectrophotometer (Thermo Scientific, Wilmington, DE, USA). The RNA quality was assessed by checking the RNA integrity numbers (RIN) and samples with similar RIN were used for all groups. Extracted RNA was stored at −80 °C until analysis.

In order to remove genomic DNA contamination, 50 ng RNA per reaction was DNAse treated (RQ1 RNase-free DNase, Promega, Dübendorf, Switzerland) in accordance with the manufacturer’s instructions. Afterwards, RNA was reverse transcribed into complementary DNA (cDNA) using Sensiscript ^™^ Reverse Transcriptase (Qiagen) and random hexamers (Applied Biosystems, Foster City, CA, USA) as primers according to the manufacturer’s instructions. All reactions were carried out in an Eppendorf Mastercycler (Vaudaux-Eppendorf AG, Basel, Switzerland).

Semi-quantitative real-time (TaqMan) PCR reactions for ERα (ESR1), ERβ (ESR2), PGR, PRL, PRLR, GHR and CDH-1 were performed as described previously [56, 57] using three independent endogenous reference genes (canine GAPDH, 18SrRNA and cyclophilin A) in the comparative CT method (ΔΔCT method). Samples were run in duplicates on a 96-well optical plate using an automated fluorometer ABI 7500 Fast Real Time PCR System (Applied Biosystems). Per reaction, 6.25 μl Fast Start Universal Probe Master (ROX) (Roche Diagnostics, Rotkreuz, Switzerland), 300 nM of each primer, 200 nM TaqMan probe, 1.75 μl autoclaved water, and 2.5 μl of 50 ng RNA were used as the TaqMan PCR reaction mixture. Amplification was performed by denaturation for 10 min at 95 °C, 40 cycles at 95 °C for 15 sec and 60 sec at 60 °C. Autoclaved water and the RT minus control were used as negative controls. The sequences of the primers and the 6-carboxyfluorescein (6-FAM) and 6-carboxytetramethyl-rhodamine (TAMRA) labeled TaqMan probes were designed using Primer Express Software (Version 2.0, Applied Biosystems) and are listed in Table 1. All primers and probes were purchased from Microsynth (Microsynth AG, Balgach, Switzerland). The canine–specific TaqMan Gene Expression Assay of Cyclophilin A (Prod. No. Cf03986523-gH), GHR (Prod No. Cf02623459_m1) and CDH-1 (Prod. No. Cf02624268_m1) were purchased from Applied Biosystems. The specificity of selected PCR products for each gene was confirmed by commercial sequencing (Microsynth).

**Table 1:** List of primers and TaqMan probes used for the semi-quantitative real-time (TaqMan) PCR

Primer	Accession number	Primer sequence	Product length (bp)
ERα (ESR1)	XM533454	Forward: 5’-CCC ATG GAG GAG ACA AAC CA-3’,	93
		Reverse: 5’-CCC TGC CTC CTC GGT GAT ATA-3’	
		TaqMan probe: 5’-CAC GGG CCC AAC TTC ATC ACA TTC-3’	
ERβ (ESR2)	XM861041	Forward: 5’-CCC AGC ACG CCC TTC A-3’	78
		Reverse: 5’-AAT CAT ATG CAC GAG TTC CTT GTC-3’	
		TaqMan probe: 5’-CCT CCA TGA TGA TGT CCC TGA CC-3’	
GAPDH	AB028142	Forward: 5’-GCT GCC AAA TAT GAC GAC ATC-3’	75
		Reverse: 5’-GTA GCC CAG GAT GCC TTT GAG-3’	
		TaqMan probe: 5’-TCC CTC CGA TGC CTG CTT CAC TAC CTT-3’	
PGR	NM_001003074	Forward: 5’-CGA GTC ATT ACC TCA GAA GAT TTG TTT-3’	113
		Reverse: 5’-CTT CCA TTG CCC TTT TAA AGA AGA-3’	
		TaqMan probe: 5’-AAG CAT CAG GCT GTC ATT ATG GTG TCC TAA CTT-3’	
PRL	NM_00108275	Forward: 5’-CAA GCC CAA CAG ATC CAC CAT-3’	104
		Reverse: 5’-ATC CCC CGC ACT TCT GTG A-3’	
		TaqMan probe: 5’-CTG AGG GTG CTG CGC TCC TGG-3’	
PRLR	HQ267784	Forward: 5’-GGA TCT TTG TTG CCG TTC TTT -3’	92
		Reverse: 5’-AAG GAT GCA GGT CAC CAT GCT AT-3’	
		TaqMan probe: 5’-ATT ATG GTC GTA GCA GTG GCT TTG AAA GGC-3’	
18SrRNA	FJ797658	Forward: 5’-GTC GCT CGC TCC TCT CCT ACT-3’	125
		Reverse: 5’-GGC TGA CCG CCT TGG TTT-3’	
		TaqMan probe: 5’-ACA TGC CGA CGG GCG CTG AC-3’	

### Statistical analysis

Raw data are presented as the mean and standard deviation (SD). A *t*-test was performed to compare serum hormone levels of dogs in different reproductive states and cycle stages. The statistical software “R” version 3.0.2 [58] and the package “nlme” [59] were used for the analysis of different statistical models with receptor gene expression as the outcome variable. Due to the repeated measurements per animal, linear mixed-effects models were applied. Animal was taken as a random effect. For the analysis of receptor gene expression in mammary tissue, outcome variables of PRLR, PGR, ERα (ESR1) and ERβ (ESR2) were used with the following predictor variables in the models: corresponding hormones of PRL, P4, E2; tissue group with the categories normal, benign, malignant; reproductive state and/or cycle with the categories spayed, anestrous, diestrous. Additionally, interactions between tissue group and hormone levels were evaluated. For the outcome variables GHR and CDH-1, the following predictor variables were tested: tissue group (normal, benign, malignant), reproductive state and/or cycle stage (spayed, anestrous, diestrous). For GHR and CDH-1, interactions between tissue group and reproductive state and/or cycle stage were also analyzed. Model selection was based on the Akaike information criterion (AIC) with lower values indicating a better model fit. A difference of at least −2 was considered as indicative of a better model fit. If AIC values differed by less than 2, the model with the lowest number of predictors was chosen (principle of parsimony). Results of the linear mixed effect models are given as p-values and effect size with their standard errors (SE). A p < 0.05 was considered significant.

## Results

### Animals and neoplasm classification

The ages of dogs at the time of blood and tissue sampling ranged from 4.6 to 16.4 years (mean ± SD, 9.8 ± 2.69). Bitches were of different breeds, the most frequent being mixed breed dogs (n = 9), Jack Russell terriers (n = 3), Boxers (n = 2) and dachshunds (n = 2).

Twenty-four bitches were intact, of which 13 were in anestrus and 11 in diestrus. Eight dogs had been previously spayed before the time of mastectomy, six between the ages of 7 and 10 years, one before 18 months of age, and in one dog no information was available.

The group of benign mammary neoplasms consisted of 19 tissue samples out of 12 dogs, the malignant neoplasia group of 37 samples from 24 dogs. Benign neoplasms included complex adenomas (n = 10), simple adenomas (n = 6), mixed benign mammary tumors (n = 2) and one ductal adenoma. The malignant group consisted of complex carcinomas (n = 14), simple carcinomas (n = 12), anaplastic carcinomas (n = 3), solid carcinomas (n = 3), ductal carcinomas (n = 3), one adeno-squamous carcinoma, and one carcinoma and malignant myoepithelioma. Normal tissue was available from 28 dogs.

### Hormonal analysis and gene expression

Serum levels of E2, P4 and PRL varied between 0.52 and 16.32 pg/ml, 0.05 and 91.4 ng/ml, and 1.32 and 8.91 ng/ml, respectively (Table 2). Serum for E2 measurement was not available from two dogs that were both affected by simple adenomas.

**Table 2:** Serum E2, P4 and PRL concentrations in dogs with mammary neoplasms

	E2 (pg/ml)	P4 (ng/ml)	PRL (ng/ml)
all dogs (n = 32)	7.85 ± 4.70	9.57 ± 21.50	3.97 ± 1.92
spayed (n = 8)	2.35 ± 1.12^a^	0.30 ± 0.27	2.97 ± 1.16^b^
intact (n = 24)	9.85 ± 3.79^a^	12.67 ± 24.14	4.31 ± 2.02^b^
anestrous (n = 13)	8.09 ± 4.08^c^	0.47 ± 0.40^d^	3.99 ± 2.13
diestrous (n = 11)	11.95 ± 2.05^c^	27.08 ± 30.31^d^	4.69 ± 1.90

Values are shown as mean ± SD. Same superscripts within a column denote significant differences at p < 0.05 (assessed with a *t*-test).

Gene expression of ERα (ESR1), PGR, PRLR, GHR and CDH-1 was detected in normal mammary tissues and in all mammary neoplasms, except in one carcinoma. The PRL and ERβ (ESR2) mRNA expression was below the detection limit in most of the samples in all groups, revealing more negative than positive results and, thereby, indicating their low expression levels. Corpus luteum tissue (mid-diestrus) used as a positive control tested positively for both factors (Additional file 1 (1.2)).

**Fig. 1:**
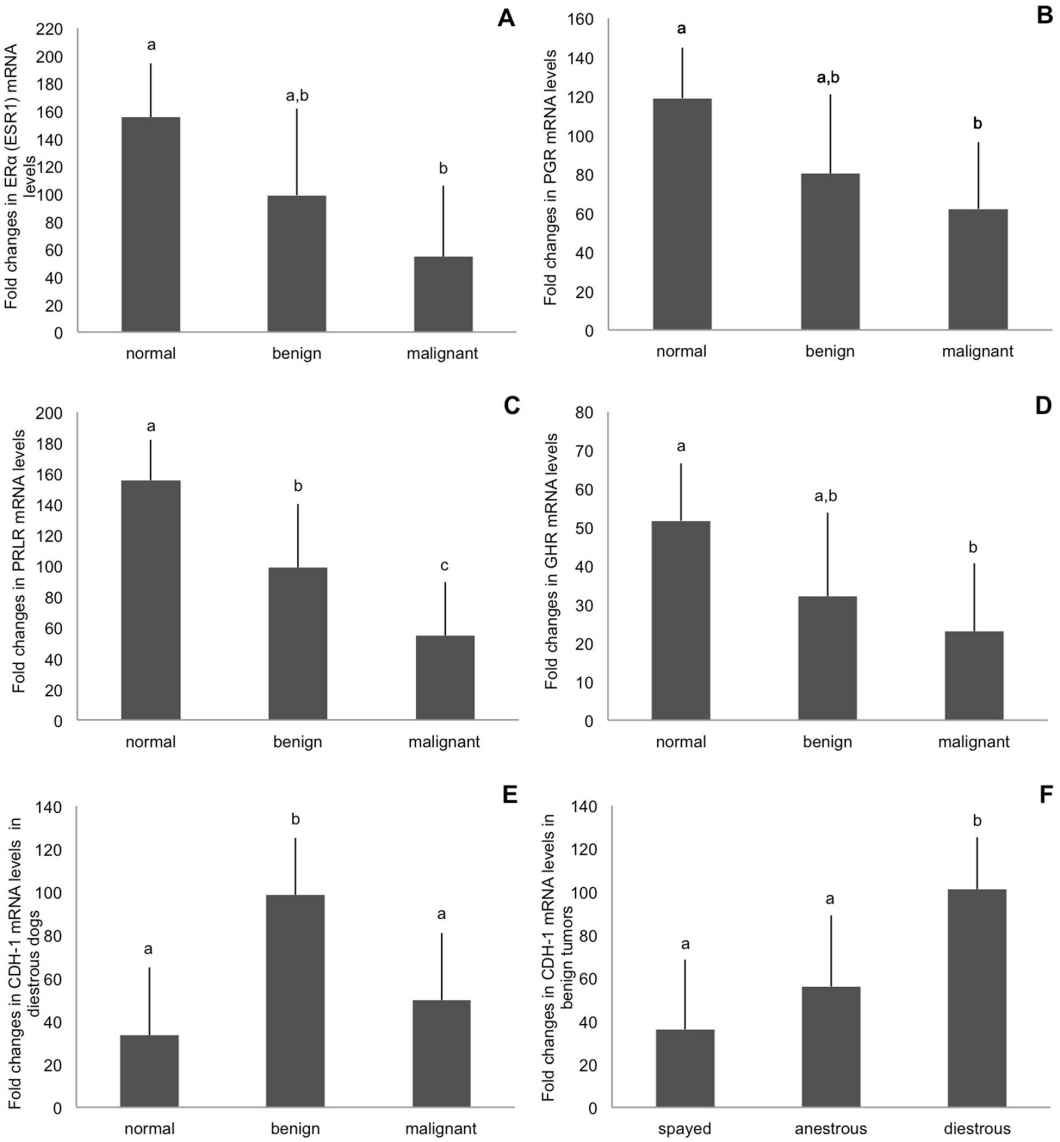
Model results of relative gene expression (fold changes in mRNA levels, mean ± SE) of **A** ERα (ESR1), **B** PGR, **C** PRLR and **D** GHR in normal mammary tissue, benign and malignant neoplasms. The significant interaction for tissue group and reproductive state/cycle on CDH-1 gene expression is shown in (**E**,**F**); **E** Relative gene expression (RGE; fold changes in mRNA levels, mean ± SE) of CDH-1 in normal mammary tissue, benign and malignant mammary neoplasms of diestrous dogs; **F** Relative gene expression (RGE; fold changes in mRNA levels, mean ± SE) of CDH-1 in benign mammary neoplasms collected from spayed dogs, anestrous and diestrous bitches. Bars with different superscripts differ at p < 0.05

Regarding gene expression of reproductive hormone receptors, the best predictor variable tested was “tissue group” for ERα (ESR1) (p = 0.03), PGR (p = 0.008) and PRLR (p = 0.001). Thus, malignant tumors showed significantly lower ERα (ESR1) and PGR gene expression than normal mammary tissues (p = 0.017 and p = 0.002, respectively; Fig. 1A and B). PRLR mRNA expression was significantly lower in both benign and malignant mammary neoplasms than in normal tissues (p = 0.009 and p < 0.001, respectively) with lowest values detected in the malignant group (Fig. 1C).

Out of the models containing “hormone blood level” and “tissue group” a relationship was only found for PRLR mRNA expression: PRL blood levels had a negative relationship with PRLR gene expression only in malignant neoplasms (p = 0.025). In contrast to these finding, the corresponding models for PGR and ERα (ESR1) did not show a relationship with their hormone levels (p = 0.07 and p = 0.11, respectively).

GHR gene expression was also predicted best by “tissue group” (p = 0.008). Consequently, GHR mRNA levels were significantly lower in malignant neoplasms compared to normal mammary tissue (p = 0.003; Fig. 1D).

In all other models, *i.e*., those using the predictors variable blood level, reproductive state and/or cycle stage, as well as the interaction between tissue group and reproductive state/cycle stage variables, were not significantly associated with receptor expression.

In contrast to the expressions of reproductive hormone receptors, the gene expression for CDH-1 revealed a significant interaction (p = 0.012) for both “tissue group and reproductive state and/or cycle stage”. Among diestrous bitches, CDH-1 gene expression was highest in benign neoplasms compared to normal mammary tissue and malignant neoplasms (p = 0.001 and p = 0.008, respectively; Fig. 1E). However, no such differences were found between samples collected in other reproductive states or cycle stages, either in spayed animals (p = 0.63 and p = 0.89, respectively) or in anestrous dogs (p = 0.09 and p = 0.09, respectively). Analyzing mammary samples according to “tissue group”, benign neoplasms from diestrous bitches expressed significantly higher levels of CDH-1 mRNA than benign neoplasms from spayed or anestrous dogs (p = 0.003 and p = 0.026, respectively; Fig. 1F). CDH-1 gene expression in malignant neoplasms and in normal mammary tissue was similar between diestrous and spayed (p ≥ 0.23), and between diestrous and anestrous dogs (p ≥ 0.33). The AIC values of models, which tested the relationship between the expression of CDH-1 and E2, P4, or PRL levels were higher than those determined for the model describing the expression of CDH-1 gene in dependence of tissue group and reproductive status/cycle stage. Thus, hormone levels had no influence on CDH-1 expression in our study.

Detailed information on hormone levels, reproductive states and cycle stages, and relative gene expression of receptors detected in normal tissues and/or mammary tumors per dog are presented in the Additional file 1 (1.1).

## Discussion

Growth of mammary epithelial cells is stimulated by steroid hormones [60, 61], PRL [62] and GH [7]. In dogs, most studies showed lower ERα (ESR1) as well as PGR expression in malignant compared to benign mammary tumors [29, 31, 63] or normal mammary tissue [16, 30, 29]. A tendency for decreased GHR expression was also noted, particularly in undifferentiated carcinomas [7, 8], which showed heterogeneous immunoreactivity compared to diffuse staining in normal tissue and benign tumors [8]. These studies are in accordance with our findings of decreased mRNA expression of ERα (ESR1) and PGR in malignant CMTs compared to normal tissue, although benign neoplasms were not different in these respects from either malignant or normal samples. The loss of or decreased receptor expression may be indicative of increasing resistance to hormonal stimulatory effects [64], and might serve as an indicator of malignancy as suggested before [8, 16, 29–31, 63].

The possible influence of steroid hormone levels on mammary tumors in dogs was previously investigated [16–18]. Thus, higher E2 concentrations were found both in serum as well as in tissue homogenates in cases of non-inflammatory malignant neoplasms compared to normal mammary tissue [16–18] or to benign tumors [17]. P4 levels showed a similar trend but only in tissue homogenates [18]. In contrast to these previous studies, which included only intact dogs in anestrus [16–18], our animal population represented different reproductive states and cycle stages including diestrous dogs, and various types of mammary tissue samples to take into account the multicentric nature and diversity of CMTs [65]. This approach may include possible effects of a malignant neoplasm on the remaining mammary gland, *i.e*., altering its gene expression levels or influencing sex steroid and PRL serum levels. However, to gather sufficient information, a much larger data base would be required. Furthermore, to investigate the postulated tumor-promoting effects of sex hormones during early carcinogenesis, longitudinal data would be desirable.

All of the above-cited studies evaluated either expression patterns of the respective hormone receptors, or the hormonal status of diseased dogs, and/or the local hormonal status of tumors. However, the association between these entities within one animal population has never been studied before. Consequently, the present study was initiated to establish such possible relationships. Based on the results presented herein, we infer that neoplasm type is the primary determinant of canine mammary steroid hormone receptor gene expression while other factors, including circulating steroid hormone serum concentrations, reproductive state and cycle stage, as well as interactions among the above, seem to have little or no influence. This is not surprising, because most dogs affected by mammary cancer have multiple lesions of different histological types [55, 66, 67]. Therefore, we intentionally included more than one neoplasm per dog when several lesions were present, and took the animal as a random factor into account in the statistical analyses as this best represented the affected dog population.

Considering the lack of the effect of the reproductive status and/or cycle stage, and thereby, of the hormonal status on the respective hormone receptor expression, this could be due to the physiological high variation of hormonal levels in dogs, overlapping with the limited number of samples used for our study, and the possible breed effects.

The PRL-PRLR complex plays a role in mammary tumorigenesis, as shown, *e.g*., in human breast cancer [68]. In agreement with our previous study [25], we detected decreasing PRLR gene expression levels during the course of malignant transformation, *i.e*., from normal mammary tissue towards benign and further to malignant transformation [25]. However, in that previous study, the reproductive state of the dogs was unknown, serum hormone levels were not investigated and CMTs consisted of a more homogeneous group of adenomas and adenocarcinomas. In the present study, we found increased serum PRL levels in malignant CMTs, which were associated with low PRLR gene expression. Similarly, high PRL concentrations in serum and tissue homogenates were previously shown in dogs suffering from malignant tumors [17, 23]. One possible explanation for these findings is that high levels of PRL induced down-regulation of PRLR transcription in the mammary gland. This could be a sign of loss of differentiation in the neoplastic cells, in which case PRL would have little or no effect in these malignant neoplasms. PRL is known to modulate the availability of its own receptor by stimulating its proteolytic degradation. This impaired PRLR turnover, which leads to its stabilization and accumulation, presumably increases the magnitude and duration of PRL signaling and contributes to transformation of human mammary epithelial cells [69, 70].

As indicated above, in dogs as in humans, PRL can be produced not only in the anterior pituitary, but also in normal mammary tissue and malignant mammary tumors [17, 23, 71–73]. Moreover, higher tissue PRL levels were observed by Queiroga and colleagues in CMT homogenates [17, 23]. Consequently, the autocrine/paracrine loop of mammary PRL-PRLR complex [71–73] contributing to human breast cancer development [74] has also been implied for the dog [17, 75]. Contrasting with these previous findings, in the present study the local expression of PRL mRNA was below detection limits in the canine mammary gland. However, mRNA concentrations might not always reflect the actual PRL concentrations at the protein level.

The activation of PRLR by PRL results in signaling through the Jak2/Stat5a pathway. Stat5 activation initiates cell differentiation through expression of the CDH-1-β-catenin complex [76, 77], which may suggest a protective effect of PRL against neoplasm formation, because decreased Stat5a activation was associated with metastatic progression in human breast cancer [78]. At present, it is still not clear whether PRL acts as a promoter or a suppressor of neoplasm development or as a promoter of differentiation [79–82]. Nevertheless, reduced immunohistochemical expression of CDH-1 in the mammary gland was related to malignancy, invasive growth, lymph node metastasis, necrosis, differentiation grade, size and ulceration of the tumor in dogs [45–48, 83]. There is limited knowledge on the molecular mechanisms behind CDH-1 down-regulation in CMTs, and about when it occurs during neoplastic progression. Pardini and colleagues [84] showed decreased CDH-1 mRNA expression in dogs with mammary carcinoma compared to normal tissue. We could not confirm this finding, as in our study similar mRNA concentrations were detected in normal mammary tissue and in malignant neoplasms. However, modifications in post-translational N-glycosylation of CDH-1 [85] cannot be ruled out, which may result in decreased cell-cell adhesion in the malignant lesions. Interestingly, we found increased CDH-1 gene expression in benign compared to malignant neoplasms, which supports the role of CDH-1 as a marker for cell differentiation. This is in line with the lack of metastatic and invasive properties of benign neoplasms. The highest CDH-1 mRNA levels were seen in benign lesions during the diestrus phase, which may be attributed to the effects of P4. Similarly, CDH-1 gene expression was up-regulated in the canine uterus under P4 treatment and during pregnancy [86]. In contrast to our expectations, reproductive state or cycle stage had no effect on CDH-1 mRNA levels in normal mammary tissue. This could be, however, due to the source of the normal/healthy tissue used for the present study, which was collected from dogs affected by mammary tumors. The microenvironment of malignant mammary tumor could have a modulatory effect on the expression of CHD-1.

## Conclusion

The transformation from a normal to a malignant phenotype was associated with a significant loss of ERα (ESR1), PGR, GHR and PRLR gene expression in the mammary tissue of dogs. Only PRLR gene expression was significantly decreased concurrently with the formation of benign tumors. Increased levels of serum PRL, but not of P4 and E2, were associated with a decrease in gene expression of the respective receptors only in malignant mammary neoplasms. No evidence was found for the presence of a paracrine/autocrine action of PRL in the canine mammary gland. CDH-1 mRNA expression was higher in benign compared to malignant neoplasms and normal mammary tissue and thus may serve as a prognostic marker.

## Additional file

Additional file 1:
**(1.1) Hormone levels, reproductive states, cycle stages, and relative gene expression of receptors in normal tissues and/or mammary tumor(s) per dog.** (1.2) Positive controls for luteal PRL and ERβ (ESR2) expression as determined by conventional RT-PCR. (PDF 158 kb)

## Abbreviations

HE: hematoxilin and eosin
RIN: RNA integrity numbers
RGE: relative gene expression
CMT: Canine mammary tumors
P4: Progesterone
PGR: Progesterone receptor
E2: Estradiol 17β
ERα (ESR1): Estrogen receptor α
ERβ (ESR2): Estrogen receptor β
PRL: Prolactin
PRLR: Prolactin receptor
GH: Growth hormone
GHR: Growth hormone receptor
CDH-1: E-cadherin
RIA: Radioimmunoassay
