# Supplementary material for: Endocrine control of canine mammary neoplasms: serum reproductive hormone levels and tissue expression of steroid hormone, prolactin and growth hormone receptors
Source: BMC Vet Res. 2015 Sep 15;11:235. doi: 10.1186/s12917-015-0546-y (PMC4570623; doi:10.1186/s12917-015-0546-y)
Supplement: Additional file 1: — (1.1) Hormone levels, reproductive states, cycle stages, and relative gene expression of receptors in normal tissues and/or mammary tumor(s) per dog. (1.2) Positive controls for luteal PRL and ERβ (ESR2) expression as determined by conventional RT-PCR. (PDF 158 kb) [file 12917_2015_546_MOESM1_ESM.pdf]

Additional File 1:

1.1 Hormone levels, reproductive states, cycle stages, and relative gene expression (RGE) of receptors in normal tissues and/or mammary tumor(s) per dog
N/A: not available

dog	reproductive state/ cyclus	E2 pg/ml	P4 ng/ml	PRL ng/ml	normal mammary tissue per dog (RGE)					mammary tumor(s) per dog (RGE)					
					**PRLR**	**PGR**	**ERα**	**GHR**	**CDH-1**	**tumour typ**	**ERα (ESR1)**	**PGR**	**PRLR**	**GHR**	**CDH-1**
1	intact (diestrus)	N/A	3.69	4.48	55.18	92.15	N/A	9.29	20.25	simple adenoma	N/A	223.13	115.53	13.29	130.43
2	intact (anestrus)	11.40	0.13	3.17	148.55	312.11	76.95	33.08	53.47	complex adenoma	30.84	72.45	90.51	9.54	53.01
										complex adenoma	13.77	7.31	26.16	8.22	40.48
3	intact (diestrus)	11.53	10.03	8.94	79.51	10.78	123.29	3.03	10.56	simple adenoma	46.58	173.17	114.53	8.56	72.17
										complex adenoma	73.90	118.35	135.57	18.24	77.64
4	intact (anestrus)	N/A	0.26	4.30	282.09	221.89	N/A	274.77	49.08	simple adenoma	N/A	180.66	255.83	25.05	31.49
										simple adenoma	N/A	55.45	78.23	18.10	77.74
5	intact (anestrus)	9.46	1.13	3.20	99.99	128.35	239.54	125.79	73.77	complex adenoma	46.94	66.67	114.75	123.67	42.12
6	spayed	2.35	0.22	3.41	147.38	102.97	29.47	59.55	64.14	complex adenoma	297.08	88.35	108.75	56.16	32.59
7	spayed	1.87	0.1	1.32	233.58	56.68	139.59	99.69	46.13	simple adenoma	439.53	159.43	223.74	63.70	97.05
										complex adenoma	296.24	38.42	130.94	80.08	56.70
										mixed benign mammary tumor	109.35	16.78	51.47	51.71	37.29
8	intact (anestrus)	5.43	0.11	8.05	218.99	132.99	159.00	4.87	36.49	anaplastic carcinoma	31.67	30.71	8.14	5.38	18.85
9	intact (anestrus)	16.32	0.07	2.02	155.48	71.66	230.37	54.00	38.72	complex carcinoma	14.74	29.33	17.95	27.24	14.55
										complex carcinoma	6.69	42.30	24.36	10.40	40.12
10	spayed	0.52	0.05	2.89	100.99	52.76	29.39	40.23	60.72	simple carcinoma	19.57	7.79	21.07	8.12	7.43
										complex carcinoma	3.71	8.90	22.41	11.31	45.92
11	intact (diestrus)	13.23	51.1	3.34	N/A	N/A	N/A	N/A	N/A	simple carcinoma	116.18	146.50	110.45	72.13	56.73
12	intact (anestrus)	4.27	0.67	2.04	67.53	124.34	49.01	14.26	18.95	simple carcinoma	146.58	19.12	184.20	27.68	67.45
										complex carcinoma	40.10	163.79	148.59	29.70	44.10
										complex carcinoma	99.91	126.03	87.04	14.72	27.28
13	intact (diestrus)	10.79	70.94	3.41	461.70	57.31	121.16	186.13	11.83	ductal carcinoma	29.17	62.16	77.73	34.09	28.99
14	intact (anestrus)	5.91	0.71	2.78	140.19	139.17	73.65	22.62	22.94	simple carcinoma	53.70	20.39	7.93	12.40	30.66
										adeno-squamous carcinoma	11.94	3.81	42.46	11.09	35.74
15	intact (anestrus)	13.87	0.24	3.14	90.13	46.93	47.46	13.01	9.95	simple carcinoma	5.61	20.19	27.28	5.86	18.82
										complex carcinoma	80.21	25.85	70.53	6.10	17.94
16	spayed	1.66	0.32	4.39	N/A	N/A	N/A	N/A	N/A	anaplastic carcinoma	2.86	1.19	8.54	5.97	1.00
17	spayed	4.44	0.47	1.79	77.00	213.01	2.92	80.74	39.56	complex carcinoma	2.05	89.67	224.29	43.04	45.64
18	intact (anestrus)	3.26	1.34	2.81	N/A	N/A	N/A	N/A	N/A	simple carcinoma	114.60	158.41	72.68	19.28	36.07
19	intact (anestrus)	6.40	0.08	8.23	282.40	159.01	189.22	28.69	56.43	anaplastic carcinoma	3.30	1.76	2.29	10.60	32.61
20	intact (anestrus)	8.66	0.44	2.77	197.63	148.29	80.62	12.62	37.79	simple carcinoma	79.30	129.57	72.95	7.81	82.83
21	spayed	2.63	0.15	1.97	N/A	N/A	N/A	N/A	N/A	simple carcinoma	65.46	181.29	79.54	34.28	101.04
										simple carcinoma	20.20	72.64	35.11	13.53	18.90
22	intact (diestrus)	11.70	91.4	4.63	45.24	26.47	97.62	31.57	72.61	complex carcinoma	31.80	51.33	50.76	43.62	29.28
23	intact (diestrus)	9.02	5.54	4.22	93.39	197.14	49.06	54.77	47.68	complex carcinoma	523.39	91.15	104.05	19.75	36.46
24	intact (diestrus)	13.08	18.54	7.64	264.84	33.94	15.93	2.86	49.29	carcinoma and malignant myoepithelioma	7.36	63.58	29.02	8.84	33.55
25	spayed	2.45	0.87	3.95	155.03	354.87	412.40	37.07	43.26	complex adenoma	58.90	8.82	30.10	11.39	24.45
										complex carcinoma	15.97	31.33	16.22	15.85	6.56
										complex carcinoma	87.69	96.70	59.16	24.06	60.88
26	intact (diestrus)	9.45	6.66	4.08	119.03	179.96	105.01	92.02	26.27	complex adenoma	109.78	3.33	65.00	63.99	108.02
										simple carcinoma	73.82	147.30	61.95	39.11	148.07
27	intact (anestrus)	8.05	0.39	3.07	187.36	52.72	286.29	11.25	34.33	ductal adenoma	6.68	24.91	5.47	7.80	85.92
										ductal carcinoma	6.26	30.08	14.59	4.00	55.91
28	intact (diestrus)	11.11	7.1	2.50	66.86	143.43	87.05	32.31	38.98	simple adenoma	114.20	24.81	100.59	57.66	103.97
										simple carcinoma	96.58	57.61	47.66	12.58	79.87
										complex carcinoma	2.28	12.36	31.10	2.59	34.70
										solid carcinoma	4.43	10.21	4.35	4.61	31.87

29	intact (diestrus)	15.70	8.37	4.34	194.41	46.89	54.70	9.61	31.40	simple carcinoma	9.87	7.05	32.80	8.86	15.29
										complex carcinoma	N/A	74.60	54.79	17.10	18.00
30	intact (diestrus)	13.87	24.53	4.06	123.14	21.35	69.03	27.40	24.57	ductal carcinoma	77.63	92.55	75.51	52.70	84.12
31	spayed	2.88	0.22	4.01	168.59	166.80	268.48	69.10	17.19	complex adenoma	111.66	196.15	193.50	76.61	37.47
										complex adenoma	28.75	43.46	33.46	17.39	10.09
										benigne mixed tumor	23.75	20.52	6.11	19.67	3.49
32	intact (anestrus)	4.10	0.5	6.22	99.92	36.35	80.65	7.96	18.19	complex carcinoma	39.62	62.72	30.25	21.44	20.90
										solid carcinoma	NA	7.74	N/A	3.70	64.86
										solid carcinoma	9.65	117.36	1.60	2.33	46.02

1.2 Positive controls for luteal PRL and ERβ (ESR2) expression as determined by conventional RT-PCR

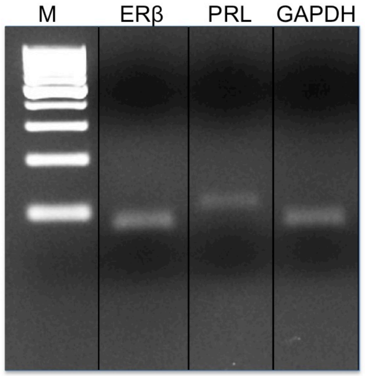

GAPDH = reference gene

M = molecular weight marker
